# Ontogenetic Variation of the Human Genome

**DOI:** 10.2174/138920210793175958

**Published:** 2010-09

**Authors:** Y.B. Yurov, S.G. Vorsanova, I.Y. Iourov

**Affiliations:** 1 Institute of Pediatrics and Children Surgery, Rosmedtechnologii; 2 National Research Center of Mental Health, Russian Academy of Medical Sciences; 3 Moscow City University of Psychology and Education, Moscow, Russia

**Keywords:** Ontogeny, somatic genome variations, chromosome instability, prenatal development, aging, aneuploidy, genomic instability.

## Abstract

The human genome demonstrates variable levels of instability during ontogeny. Achieving the highest rate during early prenatal development, it decreases significantly throughout following ontogenetic stages. A failure to decrease or a spontaneous increase of genomic instability can promote infertility, pregnancy losses, chromosomal and genomic diseases, cancer, immunodeficiency, or brain diseases depending on developmental stage at which it occurs. Paradoxically, late ontogeny is associated with increase of genomic instability that is considered a probable mechanism for human aging. The latter is even more appreciable in human diseases associated with pathological or accelerated aging (i.e. Alzheimer’s disease and ataxia-telangiectasia). These observations resulted in a hypothesis suggesting that somatic genomic variations throughout ontogeny are determinants of cellular vitality in health and disease including intrauterine development, postnatal life and aging. The most devastative effect of somatic genome variations is observed when it manifests as chromosome instability or aneuploidy, which has been repeatedly noted to produce pathologic conditions and to mediate developmental regulatory and aging processes. However, no commonly accepted concepts on the role of chromosome/genome instability in determination of human health span and life span are available. Here, a review of these ontogenetic variations is given to propose a new “dynamic genome” model for pathological and natural genomic changes throughout life that mimic those of phylogenetic diversity.

## INTRODUCTION

Natural variation in the genome at DNA sequence and chromosome level is a pervasive feature of eukaryotes underlying phylogenetic diversity of chromosome structure and numbers among species during evolution [1, 2]. Similar processes seem to occur during ontogeny in the same organism and commonly manifest as chromosome instability (CIN) or aneuploidization [3]. In humans, CIN/aneuploidization affects almost all conceptions and achieves the highest rate in day 3 or preimplantation embryos or the first trimester of prenatal development [4-7]. At the following developmental stages, the rates seem to decrease significantly either through programmed cell death or through spontaneous aborting. This leads to at least threefold decrease of instability rates after birth [5, 8-10]. However, persisting throughout cellular divisions probably due to failed clearance of affected cells, such instabilities may give rise to pathological conditions. More precisely, CIN manifesting as somatic aneuploidy is associated with abnormal prenatal development, chromosomal syndromes, cancer, and brain diseases [8, 9, 11-18]. Therefore, acquired genomic instability (GIN) initiated by CIN/aneuploidization can be considered as a regulatory developmental process and, if failed, a mechanism for human prenatal mortality and postnatal morbidity. Additionally, related manifestations of GIN and CIN are consistently observed to hallmark human aging and are suggested to result from exhausting of mitotic machinery [19-22]. The latter, however, remains uncertain, because GIN, CIN and aneuploidzation are also observed in aged tissues, which are composed of post-mitotic cells (i.e. brain). Recently, a hypothesis has been developed to solve this paradox through changing current assessing of time-scale of biological processes with new twists on GIN/CIN effect on cellular homeostasis [22]. Nonetheless, there are no available and commonly accepted concepts on the role of genetic instabilities in determination of human health or life span. To fill this gap, a review of ontogenetic variation of the human genome appears to be required.

## SOMATIC GENOME VARIATIONS (SGV) DURING PRENATAL DEVELOPMENT

There are numerous evidences for high rates of SGV in human embryos. Up to 50% of human conceptions appear to be aneupoid, representing, therefore, cases of unstable genome due to meiotic nondisjunction [3, 8, 9, 23-25]. However, the overwhelming majority of aneuploid embryos seem to be spontaneously aborted at the following developmental stages [3, 8, 26]. Nonetheless, studying of preimplantation embryos that demonstrates genomic variations at the next ontogeny stages depicts occurrence of SGV in almost all specimens affecting no less than 20-50% of cells [3, 8, 23, 24, 27]. The latter is supposed to manifest essentially as aneuploidy, but more recent reports have shown other types of CIN (structural genomic rearrangements) to be involved, as well [7]. Consequently, the instabilities are hypothesized to diminish through the prenatal development. Otherwise, a pathological condition is likely to occur [3, 8, 22]. Unfortunately, no direct evidences for this are, as yet, available, but analysis of spontaneous abortions and brain diseases in children supports these assumptions [8, 13, 15, 28, 29]. Another feature of CIN during early ontogeny stages is the ability to confine to a tissue. The best documented in this context is chromosomal mosaicism confined to placenta that is identified in about 1-2% of all the conceptions by prenatal diagnosis [30]. Another documented cause of tissue-specific chromosomal mosaicism is the confinement of aneuploidy to the developing human brain [6]. Fetal ovarian tissues seem to exhibit increased rates of aneuploidy involving chromosome 21, as well [25]. Additionally, non-random tissue-specific distribution of karyotypically abnormal cells is observed in prenatally analyzed cases of supernumerary marker chromosomes [31]. Table **1** summarizes available data on developmental CIN in humans. These observations evidence that a variety of CIN-associated processes does occur in human fetuses and suggest a definitive role of SGV in human prenatal development. CIN manifesting as aneuploidy appears to be involved in the normal human placentation [32]. As to the establishment of the role that developing CIN and aneuploidization plays, one can propose to compare CIN rates before and after birth.

## SGV DURING POSTNATAL LIFE: LATE ONTOGENY AND AGING DISEASES

Unfortunately, data on SGV in the early postnatal life of unaffected individuals is almost unavailable. Nevertheless, controls in studies of brain diseases in childhood exhibit insignificant rates of mosaic aneuploidy and CIN [13, 18, 33]. A comparison of sporadic aneuploidy rates in the developing and adult human brain shows an exact threefold decrease [5, 6, 8, 10, 16, 18, 33]. Otherwise, related studies were performed for the clinical population only [3, 8, 9, 15, 34, 35]. Human aging tissues appear to be more thoroughly studied in this context. Therefore, there is a gap in our knowledge about ontogenetic variations of the human genome in childhood and early adulthood. However, to define whether GIN or CIN increases during human life, control population, that is usually individuals of adult or middle age, has been analyzed [20, 22, 36-39]. Another possibility to prove the existence and effects of age-related SGV is referred to studying diseases associated with accelerated or pathological aging [22]. Currently, there are more than 30 diseases that are associated with GIN, CIN and aging. However, only few of them were evaluated in the light of genome variations manifesting as GIN or CIN. Among these are some monogenic CIN syndromes, Alzheimer’s disease (AD) and Down syndrome (trisomy of chromosome 21) [18, 22, 33, 40, 41]. Additionally, aneuploidy, as a whole, is likely to cause accelerated senescent phenotypes either at cellular or at organismal level [17, 22, 42-45]. The latter is also observed in cancer-predisposing diseases (i.e. ataxia-telangiectasia or AT) and in malignant tissues [18, 21, 33, 40, 41]. Table **2** summarizes natural genome variations through human aging and GIN/CIN in aneuploidy-associated and aging diseases.

As one can see (Table **2**), regardless of the stage of ontogeny, an increase of GIN and CIN causes aging phenotypes. However, normal ontogenetic variations of the human genome are more apparent when aging tissues are studied [5, 10, 16, 18, 20, 22, 36-39]. This raises an important question about their origins. Firstly, GIN and CIN (aneuploidization) cause a wide spectrum of diseases both in childhood and in adulthood [8, 9, 11-18, 26, 29, 33-35]. Additionally, some GIN/CIN signatures such as uniparental disomy due to intrauterine trisomy rescue *via* confined placental mosaicism [46] and low-level chromosomal mosaicism in child and adult individuals [3, 8, 9, 34] have been reported. Taken together, it suggests previous (GIN’n’CIN) hypothesis applied to the human brain [22] to be also true for all the human tissues. Thus, the timeline of ontogenetic genomic variations mediated by GIN, CIN or aneuploidization is as follows: CIN achieves the highest rate during early perinatal development, then GIN/CIN signatures are only observed (apart from pathologic conditions) and, finally, it begins to increase in late ontogeny. This appears to fit well data on SGV in different human tissues and implicates mitotic machinery exhausting as the main cause of aging-associated GIN/CIN progression [19, 22]. However, post-mitotic cells are unlikely to become aneuploid *via* these mechanisms [22]. Nevertheless, molecular cytogenetic replication analyses that depict mitotically non-arrested cells [47-49], yielded positive results in the diseased brain [50]. Further studies have demonstrated gene mutations associated with early-onset AD to be involved in mitotic chromosomal missegregation leading to CIN [44, 45]. Despite of chromosome 21-aneuploidization of the AD brain [18], this hypothesis can still be challenged, because of highly specific CIN manifestation and technical limitations of single-cell molecular cytogenetic replication analyses. To solve this paradox, positive data from all the molecular cytogenetic studies of the AD brain and AD models have been gathered [51]. This resulted in a model proposing that accumulation of GIN and CIN due to abnormal mitotic behavior of brain cells during prenatal development and early childhood followed by natural selection leads to persistence of chromosome 21-aneuploid cells, which produce recognizable disease phenotype in the late ontogeny [22, 51]. This accords well with observations of monogenic AD models [44, 45]. Similar processes seem to underlie AT pathogenesis with the sole exception that GIN/CIN manifests in the early childhood, probably due to specifity of genome maintaining protein behavior encoded by the AT-causing gene (*ATM*) as well as more pathogenic value of CIN [18, 33]. However, one has to keep in mind that GIN and CIN in aging diseases are evaluated after manifestation. Therefore, no data is available on previous ontogenetic periods. Still, to get an integrated view of somatic genome changes throughout ontogeny, data on origins of genomic variations (somatic, germline or syntenic), their associations with aging phenotypes and parental development can be used.

## ONTOGENETIC VARIATIONS OF THE GENOME: PHYLOGENY-ONTOGENY PARALLELS AND UPDATED “GIN’N’CIN” HYPOTHESIS

**Fig. (1):**
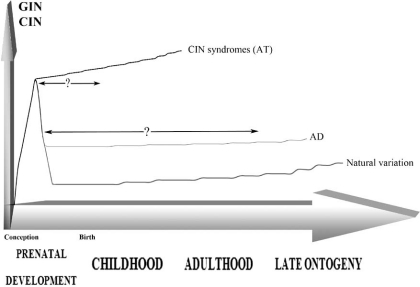
Schematic representation of SGV manifested as GIN/CIN changing during ontogeny due to natural variations of the genome and in aging diseases: AT (CIN syndrome) and AD. Being increased in the early prenatal development, GIN/CIN decreases throughout following ontogeny stages, showing, however, a slight increase in the late ontogeny. In cases of CIN syndromes, GIN/CIN probably decreases insignificantly and demonstrates high rates after birth as exemplified by studying the AT brain [17, 32]. AD is suggested to be associated with failed clearance of cells affected by GIN/CIN exhibiting high rates of the chromosome 21 aneuploidy in diseased brain analyzed after the manifestation (late ontogeny) [7, 17, 21]. Intervals marked by question symbol show ontogenetic periods that have not been studied in context of SGV.

Summarizing the data described above, we have proposed a schematic graph showing the trend of genome variation throughout ontogeny due to natural intercellular genome variations and in aging diseases associated with GIN and CIN (Fig. **1**). The graph also indicates ontogenetic stages that need to be analyzed for definition of mechanisms by which SGV are formed and maintained throughout development to produce the aging phenotype (at least in AT and AD). As to natural ontogenetic genome variations, the dynamics appear to be better understood [3, 8]. However, this has to be thoroughly re-evaluated and the data on benign SGV have to be provided. 

Since the evaluation of SGV evidences that the human genome is subjected to dynamic changes during ontogeny, it appears appropriate to refer to “dynamic genome”, a term introduced to define the process of transposition of mobile genetic elements changing the amount of DNA in the genome. Discovered more than 50 years ago by Barbara McClintock, it was used to explain numerous genetic processes including genome changes during phylogeny [52]. Current concepts in genome research allow to extrapolate principles of phylogenomics to SGV [3, 53]. This is, in parts, inspired by the observations of parallels between chromosome number/structure or DNA sequence variations along branches of the phylogeny and somatic mutations manifesting as GIN or CIN along ontogeny [3, 22, 53-56]. If phylogenomic principles are applied to cellular genomes, Darwinian or natural selection appears to play a role not only in evolution, but also in brain diseases and cancer [3, 53, 54, 56]. As one can see, it can be also successfully applied to the normal human development and aging (ontogeny), as well [3, 6, 9, 22, 53, 54]. Therefore, during prenatal development, aneuploidization or CIN/GIN serves as a mechanism for regulation of cell population size through the clearance of abnormal cells or growth arrest [6, 8, 22, 32, 51, 57]. The lack of clearance or increase of cell viability due to genomic variations (as in malignant cells) would lead to pathological conditions such as aneuploidy-associated diseases or cancers. In the late ontogeny, increase of CIN/GIN would lead to aging phenotype due to either a failure of abnormal cell clearance or aging-related alterations to mitotic machinery. The aging tissues composed of post-mitotic cells seem to become senescent by a different mechanism that is probably related to changes of genome expression in cells with abnormal chromosome complement accumulated at earlier ontogenetic stages. To this end, “dynamic genome” model in the ontogenetic sense can be postulated as follows: SGV in early prenatal development reach its highest rate in order to highlight cells for cell clearance machinery (this makes a decrease of abnormal cell content); at the following ontogenetic stages, the amount of cells with altered genome exhibits less significant variation, which is undetectable by available techniques; in the latest ontogenetic stages, a re-increase of SGV rates is observed and is associated with aging; finally, the rates of ontogenetic genome variations are probably specific for each tissue.

## CONCLUDING REMARKS

Currently, almost all the studies in the fields of human genetics and genomics operate with “an average cell genome”. The approach allows the determination of genomic variations between individuals. Although it is unavoidable to apply such technologies for genomic studies, SGV cannot be adequately described by techniques analyzing DNA isolated from a large pool of cells. As a result, much less attention is paid to such phenomena as ontogenetic variations of the human genome, which are impossible to evaluate by studying “average cell genome”. Single-cell genomic as well as single cell proteomic approaches are more sophisticated [3, 15, 34, 58] and their results are much more difficult to interpret. Nevertheless, several molecular cytogenetic studies demonstrated the existence of SGV and their role in normal human development, disease and aging. Regardless of available data, which allows to propose hypotheses and models describing genome variations over the human lifespan, ontogenetic genome research requires additional studies. Taking into account that genome variations are likely to be specific for each ontogenetic stage and each tissue, one can imagine a large amount of work still to be performed. The present model and an update of GIN’n’CIN hypothesis intend to shed light on the genome behavior during ontogeny in health and disease. The future of genomic research appears to be linked with single-cell biology that refutes the “average cell concept” and postulates a continuous (structural and functional) variation of cellular genome. Therefore, further studies of the human genome would be incomplete without addressing its ability to dynamically change along ontogeny.

## Figures and Tables

**Table 1.** CIN in Human Conceptions During the Prenatal Development

Developmental Stage	Tissue	Type of CIN	Normal Variation	Pathological Condition	Key Refs
Preimplantation embryos	NA	Aneuploidy	+	+	[21, 24, 27]
	NA	Aneuploidy/structural abnormalities	+	?	[7]
Fetuses at 7-12 weeks gestation	Chorionic villi	Aneuploidy	+	-	[6]
	Brain	Aneuploidy	+	?	[5, 6]
	Skin	Aneuploidy	+	-	[6]
	Ovarian tissue	Aneuploidy	+	-	[25]
Spontaneous abortions (7-15 weeks gestation)	Chorionic villi	Chromosomal mosaicism (aneuploidy)	-	+	[28]
Prenatal diagnosis (~7-12 weeks gestation) (choriocentesis)	Chorionic villi Placenta	Chromosomal mosaicism (aneuploidy)	+	-	[30]
Prenatal diagnosis at 20^th^ week gestation	14 different tissues	Derivative chromosomes (marker chromosomes)	-	+	[31]

NA — not applicable.

**Table 2.** GIN and CIN (SGV) Associated with Normal Human Aging and Aging Diseases

Condition	Tissue/Cell Types	Overview of Instability	Key Refs
Natural genomic variations during normal aging			
Normal aging	Blood lymphocytes	Chromosome X: 1.5%-2.5% and 4.5%-7.3%*; Chromosome Y loss: 0.17%; Autosomes: 1.2% and 1-2%*	[20, 36, 37, 39]
	Skin fibroblasts	2,2% and 4,4%*	[38]
	Brain	0.3-0.9% and 1.4-3%* (no targeted studies of the aging brain are, as yet, available)	[5, 10, 16, 18, 22, 33]
**Aneuploidy and aneuploidy-associated/aging diseases**			
Aneuploidy	Aneuploid cell lines	Aneuploid cells demonstrate senescent phenotypes	[42]
Down syndrome(trisomy 21)	Blood lymphocytes (other tissues are rarely analyzed)	100% (?) of cells with additional chromosome 21 cause accelerated aging phenotype	[17, 25, 43]
AT	Brain	Aneuploidy and chromosome breakage producing additional rearranged chromosomes (partial aneuploidy) confine to the degenerated cerebellum and affect up to 40% of cells	[18, 33]
AD	Brain	Chromosome 21 aneuploidy affecting 6-15% of brain cells	[18]
	Transfected human presenelin1-mutated cells	Acquired chromosome missegregation causing aneuploidy associated with abnormal presenelin 1 functioning	[44]
	Transgenic mice and transfected human cells	Amyloid precursor protein gene (APP) induce chromosome missegregation and aneuploidy	[45]
Cancers	Almost all types of malignant tissues/cells	Aneuploidy hallmarks almost all cancers; aneuploid cells exhibit senescent phenotype	[14, 21, 40, 41]

* — middle age and aged individuals, respectively.
